# Successful use of extracorporeal membrane oxygenation in a human immunodeficiency virus infected patient with severe acute respiratory distress syndrome

**DOI:** 10.1186/1742-6405-11-37

**Published:** 2014-11-21

**Authors:** Robertas Samalavicius, Mindaugas Serpytis, Donata Ringaitiene, Daiva Grazulyte, Ruta Bertasiute, Bernardas Rimkus, Raimonda Matulionyte, Ruta Ambrazaitiene, Jurate Sipylaite, Tomas Kacergius, Laimonas Griskevicius

**Affiliations:** Center of Anaesthesiology, Intensive Care and Pain Management, Vilnius University Hospital Santariskiu Klinikos, Vilnius, Lithuania; Clinics of Anaesthesiology and Intensive Care, Faculty of Medicine, Vilnius University, Vilnius, Lithuania; Hematology, Oncology and Transfusion Medicine Center, Vilnius University Hospital Santariskiu Klinikos, Vilnius, Lithuania; Clinics of Internal, Family Medicine and Oncology, Faculty of Medicine, Vilnius University, Vilnius, Lithuania; Department of Infectious, Chest diseases, Dermatovenerology and Allergology, Faculty of Medicine, Vilnius University, Vilnius, Lithuania; Center of Infectious Diseases, Vilnius University Hospital Santariskiu Klinikos, Vilnius, Lithuania; Center of Laboratory Medicine, Vilnius University Hospital Santariskiu Klinikos, Vilnius, Lithuania; Department of Physiology, Biochemistry, Microbiology and Laboratory Medicine, Faculty of Medicine, Vilnius University, Vilnius, Lithuania

**Keywords:** HIV, veno-venous ECMO, ARDS, multidrug resistant bacteria, VAP

## Abstract

**Introduction:**

We report a case of an adult patient with human immunodeficiency virus (HIV), acute respiratory distress syndrome (ARDS) and ventilator associated pneumonia (VAP) caused by multidrug resistant (MDR) bacteria that was successfully managed with veno-venous extracorporeal membrane oxygenation (ECMO).

**Case report:**

A 25 year old male with no significant past medical history had been admitted to a local hospital due to dyspnea and fever. His pulmonary function subsequently failed necessitating mechanical ventilation (MV) and introduction of ECMO support. The patient was transported for 300 km by road on ECMO to a tertiary medical center. The diagnosis of ARDS, HIV infection and MDR bacterial and fungal VAP was made. Patient was successfully treated with antiretroviral therapy (ART), anti-infective agents and 58 days of veno-venous ECMO support, with complete resolution of the respiratory symptoms.

**Conclusion:**

HIV infected patients with ARDS and MDR bacterial VAP whose HIV replication is controlled by ART could be successfully managed with ECMO.

## Background

According to Berlin definition [1] acute respiratory distress syndrome (ARDS) is characterized by 1) lung injury of acute onset, within 1 week of an apparent clinical insult and with progression of respiratory symptoms; 2) bilateral opacities on chest imaging not explained by other pulmonary pathology (e.g. pleural effusion, pneumothorax, or nodules); 3) respiratory failure not explained by heart failure or volume overload; 4) mechanical ventilation with decreased PaO_2_/FiO_2_ (ratio of partial pressure of arterial oxygen to fraction of inspired oxygen).

Extracorporeal membrane oxygenation (ECMO) is salvage therapy for selected group of patients with gas-exchange failure refractory to mechanical ventilation (MV). Severe hypoxemia despite the application of high levels of PEEP or uncompensated hypercapnia are the main indications for ECMO treatment. Mechanical ventilation at high settings (FiO_2_ 90%, Pplat >30 mmHg) for more than 7 days, immunosupression and intracranial hemorrhage are the main contraindications for using this mode of treatment. ECMO is associated with considerable risks for the patient, including but not limited to bleeding, infection or mechanical failure of the ECMO circuit [2]. Recently, ECMO has been shown to be very effective in treating ARDS patients during H1N1 pandemic [3] and successful use of ECMO in immunocompromised patients has been reported [4, 5]. However, it remains a relative contraindication to extracorporeal life support.

The purpose of this report is to describe an human immunodeficiency virus (HIV) infected patient with ARDS complicated by multidrug resistant (MDR) bacterial and fungal ventilator associated pneumonia (VAP) who was successfully managed with veno-venous ECMO and off-label doses of antimicrobial agents.

## Case presentation

A 25 year old male with no significant past medical history was admitted to a local hospital due to dyspnea and fever of 39°C. The patient’s pulmonary computer tomography (CT) scan was consistent with alveolitis. Sputum and blood cultures were negative. Patient received empirical Amoxicillin prior to hospitalisation and Cefuroxime in hospital without any improvement. For suspected allergic alveolitis prednisolone 40 mg daily for 8 days was administered. As a result of worsening of respiratory function on day 10 of initial hospitalization, the patient was transferred to intensive care unit (ICU). 1 gram of methylprednisolone once, followed by three consecutive doses of 120 mg/per day were administered. Pulmonary function deteriorated and mechanical ventilation was initiated on day 6 in intensive care unit stay. No signs of any other organ system dysfunction were evident. Antibiotic therapy was changed to Imipenem and Vancomycine. On day 11 of MV, the patient’s respiratory function deteriorated substantially. The decision was made to transfer the patient to Vilnius University Hospital Santariskiu Klinikos (VUHSK) with facilities for ECMO treatment.

When transport team arrived, tidal volume was decreasing to critical levels trying to achieve safe ventilation pressures, the patient had drained pneumothorax, paO_2_/FiO_2_ of 84 mmHg on 85% of FiO_2_ and 16 cm H_2_O of PEEP. As well patient had permissive hypercapnia with CO_2_ of 87 mmHg; Murray score was 4 (up to four points in each of four categories; PaO_2_/FiO_2_, number of quadrants with alveolar consolidation, PEEP, and compliance) [6]; pulmonary compliance was only 4 ml/cm H_2_O; with pressure control ventilation of 30 cm H_2_O the inspired tidal volume was only 2 ml/kg. The decision was made to place the patient on veno-venous ECMO before the transfer because paO_2_/FiO_2_ of less than 80 mmHg on 90% of FiO_2_ and Murray score of 3–4 identifies the risk of mortality of 80% with conventional treatment and is an indication for initiation of extracorporeal life support according to the extracorporeal life support organization guidelines (http://www.elso.med.umich.edu/Guidelines.html). 23 French drainage cannula (Bio-Medicus, Medtronic Inc., Minneapolis, USA) was inserted percutaneously into inferior vena cava via femoral approach and 19 French return cannula was inserted into the left internal jugular vein. ECMO circuit consisted of bioline coated Quadrox PLS oxygenator and Rotaflow centrifugal pump (Maquet Cardiopulmonary AG, Rastatt, Germany). The patient was uneventfully transported for 300 km (200 minutes) by road ambulance to VUHSK, ECMO flow was 6 l/min.

**Figure 1:**
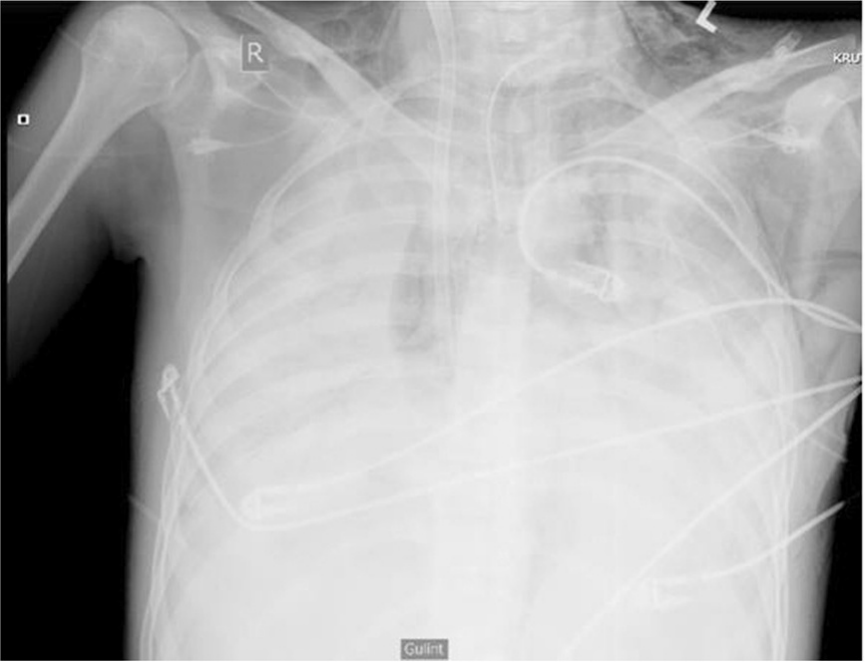
**Chest x-** **ray on admission at VUHSK** **(3 hours after ECMO start)** **.**

On admission to VUHSK, the patient’s lung compliance was 3–4 ml/cm H_2_O, his respiratory tidal volume was only 1–2 mL/kg to keep the ventilation pressure safe at <30 cm H_2_O. The patient had lung infiltration of 4 quadrants seen on chest x-ray (Figure 1). The white blood cell (WBC) count was 8.72 × 10^9^/L (neutrophils 7.71 × 10^9^/L, lymphocytes 0.66 × 10^9^/L), platelets 257 × 10^9^/L, hemoglobin 109 g/l, lactate 1.36 mmol/L and C-reactive protein (CRP) was 93 mg/L. On the first day at VUHSK, the patient was confirmed HIV positive (positive anti-HIV ½ Ag/Ab, Western Blot analysis showed three bands (p41, gp120 and p51) and HIV-1 RNA was 2.22 million copies/mL), his CD4 cell count was 134 cells/μL. The blood sample was positive for CMV DNA (442420 copies/mL). The bronchoalveolar lavage (BAL) fluid WBC count was 1.17 × 10^9^/L (99% neutrophils) and was positive for CMV DNA (76740 copies/mL), Candida glabrata and MDR Acinetobacter baumanii. The diagnosis of ARDS, HIV infection, nosocomial VAP and possible CMV pneumonitis was made. Colistin, Linezolid, Imipenem, Ganciclovir and Anidulafungin were started. ART consisting of Kivexa (Abacavir and Lamivudine) and Kaletra (Lopinavir and Ritonavir) was initiated. The patient was continued with MV, full intensive care and intermittent vasopressor support. Blood flow during ECMO was tailored to achieve acceptable oxygenation. Ventilator settings were reduced to protective ventilation mode. During the ICU stay, aggressive treatment of numerous nosocomial infections was required (Table 1). Complications during ECMO are detailed in Table 2.

**Table 1:** **Nosocomial infections and treatment**

ICU day	Bacteria				Fungus		Virus	CRP, mg/L	Treatment	MIC, mg/L	Administration route	Daily dose
	Acinetobacter baumanii*	Pseudomonas aeruginosa**	***Pseudomonas fluorescens***	***Enterobacter aerogenes***	***Candida glabrata***	***Candida parapsilosis***	***Cytomegalovirus*** ***(CMV)***					
1	BAL/CAT				BAL/CAT		BAL/BL	93	*Colistin*	1	i.v.	13.5 m. IU
									*Ganciclovir*	-	i.v.	0.8 g
3	SP							165	*Colistin*	1	i.v.	13.5 m. IU
8					BAL			77	*Anidulafungin*	0.125	i.v.	0.1 g
11	BAL							151	*Colistin*	1	i.v.	13.5 m. IU
16		BAL						286	*Ciprofloxacin*	0.5	i.v.	1.2 g
24		BAL/BL						22	*Ciprofloxacin*	0.5	i.v.	2.4 g
									*Colistin*	2	i.v./inh	13.5/6 m. IU
28, 35, 39	BAL/ UR							149-157	*Colistin*	0.5-2	i.v./inh	13.5/9 m. IU
43	BAL/UR							162	*Ampicillin Sulbactam*	2	i.v.	36 g
51		BAL						98	*Cefepim*	4	i.v.	6 g
63	BAL							86	*Colistin*	0.5	i.v./inh	13.5/15 m. IU
65, 70						BL/ CAT		73	*Amphotericin B*	0.5	i.v.	0.08 g
75			CAT	CAT	CAT			90	*Ciprofloxacin*	0.38	i.v.	2.4 g
									*Cefepim*	4	i.v.	6 g
									*Imipenem*	1	i.v.	3 g
									*Amphotericin B*	0.19	i.v.	0.08 g
77				CAT				108	*Imipenem*	<0.25	i.v.	3 g

ICU – intensive care unit, CRP – C-reactive protein, MIC – minimal inhibitory concentration, BL – blood, BAL – bronchoalveolar lavage, CAT – catheter, UR – urine, i.v. – intravenous, inh.–inhaled, m. – million, IU – international units.

*Acinetobacter baumanii was carbapenems (*Imipenem*, *Meropenem* MIC 32 mg/L), aminoglycosyde (*Amikacin* MIC 256 mg/L, *Gentamycin* MIC 24 mg/L) and cephalosporin (*Cefepim* MIC 48 mg/L) resistant;

**Pseudomonas aeruginosa was carbapenems (*Meropenem* MIC 32 mg/L), cephalosporin (*Cefepim* MIC 12 mg/L) resistant.

**Table 2:** **Complications and their treatment during ECMO**

ICU day	Complications	Treatment/Procedures
**1-** **59**	Bilateral pneumothorax	Pleural cavity drainage
**25,** **31,** **51**	Pulmonary hemorrhage	Endobroncheal hemostasis
**38**	Tilt of left jugular vein cannula with blood loss of 2 liters	Cannula reinserted, transfusions of packed red blood cells and platelets
**46**	Blood-clot masses in pleural cavity	Open right side thoracotomy with clot removal and lung decortication
**48**	Left side tension pneumothorax with cardiogenic shock	Pleural cavity drainage, resuscitation
**52**	Bleeding from tracheostoma	Endobroncheal hemostasis
	Endrobronchial clot-masses	Bronchoscopy and clot-mass removal
**60**	Cannula associated deep vein thrombosis	Heparin, compression therapy

Despite aggressive treatment, improvement of pulmonary mechanics was slow (tidal volume with P_peak_ 30 cm H_2_O was only up to 2 ml/kg, compliance remained at 1–4 ml/cm H_2_O). For the downregulation of immune reconstitution inflammatory syndrome, intravenous methylprednisolone 1 mg/kg/dose per day for 7 days was administered on ECMO day 18 and then gradually tapered off over 15 days. This resulted in improved lung function (tidal volume increased to 4.4 mL/kg with P_peak_ 27 cmH_2_O and compliance 9–10 ml/cm H_2_O).

The first oxygenator was exchanged on day 38 after decrease in function. The patient was weaned off ECMO after 56 days. Unfortunately, 48 hours later, acute hypercapnic respiratory failure developed (pCO_2_ 190 mmHg, pH 7.05). The veno-venous ECMO was reinstituted with successful weaning after 48 hours. MV was continued for additional 18 days.

**Figure 2:**
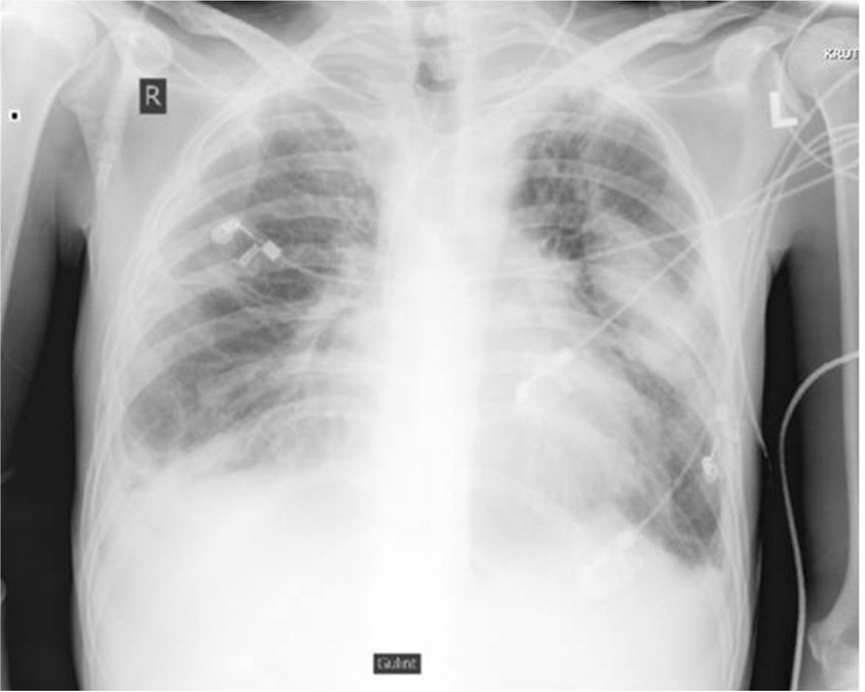
**Chest x-** **ray after weaning off ECMO.**

After 70 days of ART, the patient’s CD4 cell count increased to 268 cells/μL and the HIV-1 viral load became 591 copies/mL, lung chest X-ray is shown in Figure 2. The patient was transferred to a general ward and later discharged from hospital to continue rehabilitation. At the last clinic visit 115 days after having been discharged from ICU, the patient reported good health, had no pulmonary symptoms, could walk unassisted for more than 2 kilometers.

In total, 51 packed red blood cells and 14 platelet units were transfused. The total duration of ECMO was 58 days. The total MV duration was 87 days (11 pre ECMO, 58 ECMO and 18 post ECMO). The total VUHSK ICU stay was 84 days.

## Discussion

ECMO use in immunocompromised patients is controversial because of high mortality [7–9]. To the best of our knowledge, there is only one report of ECMO use in HIV infected patient [5]. We had not been aware of the patient’s HIV infection and thus were not biased when deciding to initiate ECMO support. Also, our patient may have had an early stage of HIV infection as witnessed by a high HIV-1 viral load in parallel with a weak antibody band spectrum on Western Blot analysis and relatively rapid CD4 count recovery.

Importantly, our patient had a predominant pulmonary failure while the function of other organs was relatively preserved making veno-venous ECMO a good choice of gas-exchange support while anti-infective therapy took effect. Also, we chose an aggressive preemptive approach to diagnose and treat nosocomial superinfections. Increased cardiac output, leaky capillaries, enlarged volume of distribution and impaired tissue penetration in septic patients may lead to an inadequate concentration of antimicrobial agent at the target site [10]. Also, due to sequestration and increased volume of distribution patients on ECMO are at risk of suboptimal antimicrobial therapy [11]. Therefore, there is a tendency to use higher doses of antimicrobial agents in critically ill patients. In our case, antibiotics were changed as soon as nosocomial bacteria were identified at doses that in many cases were considerably higher (Table 1) [12–14] than indicated in the drug label. We did not observe unacceptable antibiotic related toxicity.

ART was initiated as soon as HIV positivity was confirmed. Of note, ART suppresses HIV replication and may cause immune reconstitution inflammatory syndrome [15]. We administered a short course of methylprednisolone resulting in improved lung function. A recent meta-analysis showed a possibility of reduced mortality and increased ventilator free days with steroids in ARDS [16].

Common complications during ECMO are pneumonia, sepsis and bleeding especially associated with invasive procedures [17]. In our case, pulmonary hemorrhage, bleeding from the cannulation site and pneumothorax (including one case of tension pneumothorax) were observed necessitating surgical and medical intervention (Table 2). After first weaning off ECMO we observed hypercapnic respiratory failure leading to hemodynamic instability. This emphasizes uneven pulmonary gas exchange reconstitution with CO_2_ exchange recovery often trailing that of O_2_ [18].

## Conclusions

In individual cases, ECMO could be succesfully applied in HIV infected patients with ARDS refractory to conventional treatment and ventilator-associated pneumonia caused by multidrug resistant bacteria whose HIV replication is controlled by antiretroviral therapy. Long-distance transportation on ECMO may be attempted in selected cases.

### Consent

Written informed consent was obtained from the patient for publication of this Case report and any accompanying images. A copy of the written consent is available for review by the Editor-in-Chief of this journal.

## Abbreviations

ARDS: Acute respiratory distress syndrome
ART: Antiretroviral therapy
BAL: Bronchoalveolar lavage
CMV: Cytomegalovirus
CRP: C – reactive protein
CT: Computer tomography
DNA: Deoxyribonucleic acid
ECMO: Extracorporeal membrane oxygenation
HIV: Human immunodeficiency virus
ICU: Intensive care unit
IRIS: Immune reconstitution inflammatory syndrome
MDR: Multidrug resistant
MV: Mechanical ventilation
PEEP: Positive end-expiratory pressure
RNA: Ribonucleic acid
VAP: Ventilator associated pneumonia
VUHSK: Vilnius University Hospital Santariskiu Klinikos
WBC: White blood cell.
